# How to improve intervention research on the psychosocial work environment?

**DOI:** 10.5271/sjweh.4107

**Published:** 2023-07-01

**Authors:** Alex Burdorf

**Affiliations:** Department of Public Health, Erasmus Medical Center Rotterdam, Rotterdam, The Netherlands. [email: a.burdorf@erasmusmc.nl]

In this era of evidence-based medicine, the randomized controlled trial (RCT) has become the gold standard in determining whether a new intervention has beneficial effects. Although in recent years the RCT has given way to more flexible approaches, the strong reliance on this type of decisive study design in evidence production has had unintended consequences (1). An example in occupational health research would be that an RCT examining a health promotion app is more feasible than one assessing a workplace intervention, hence, there is disproportionately more robust evidence on individual-oriented behavioral interventions than organizational-level ones. Likewise, it would easier to evaluate a training program than an organizational intervention targeting work schedules. Indeed, a recent review on workplace mental health interventions concluded that interventions tended to focus on individual-level rather than organizational or system-level factors (2). The recent guidelines on mental health from the World Health Organization also illustrate that recommendations for individual interventions are more common than recommendations for organizational interventions (3).

In the current debate about prevention, there is a strong plea for a shift in content and mode of delivery of prevention, specifically targeting the environments in which disadvantaged groups live and work (4). While targeting these environments, the traditional RCT paradigm cannot be used as an evaluation strategy for dynamic interventions with multiple interrelated changes in behavior and environments over time (5, 6). In occupational health we face the same challenge: with mental health as a premier concern in the workforce, we need to shift our attention from individual behaviors towards the psychosocial work environment, and our research methods should change accordingly to be able to demonstrate how we can improve it.

There is compelling evidence that psychosocial work factors may introduce health effects (7). A recent meta-review of 72 reviews concluded that there is evidence for associations between high job strain and long working hours as exposures and, most notably, coronary heart diseases and (ischemic) stroke as outcomes (8). Despite many intervention studies, the insight about effective strategies to mitigate the impact of a strenuous psychosocial work environment on health is still limited. We know certainly more about the importance of the psychosocial work environment for health than we know how to improve it.

In this issue of the *Scandinavian Journal of Work Environment and Health*, Aust and colleagues (9) have undertaken the courageous effort of conducting a meta-review of 52 reviews covering 957 original studies in an effort to uncover which organizational-level interventions are effective in improving the psychosocial work environment and workers’ health. Their analytical framework has some interesting choices. First, they restricted the review to the effectiveness of organizational-level interventions at the workplace, arguing that how work is organized and conducted determines the psychosocial work environment. Hence, their focus was on primary prevention strategies to improve the work environment rather than individually targeted interventions to support individual workers in coping with psychosocial factors at work. Second, the review distinguished improvements in the psychosocial work environment (eg, increased levels of job control) from improvements in workers’ health (eg, reduced sickness absence). This distinction seems obvious, but rather surprisingly previous reviews often lack this information. This restricts our understanding about the mechanism of how an intervention can be effective.

This editorial is not the place to summarize the evidence of a meta-review, which itself summarizes the evidence of 52 reviews. Read the article yourself and be surprised by the quality and density of information on the evidence of organizational-level interventions. However, the meta-review shows some findings that should challenge the research community.

First, 28 out of 52 reviews focus on the healthcare sector. Healthcare organizations are apparently easily accessible and reachable, and have plenty of study participants. The feasibility of a research project seems more important than scientific or societal considerations. Hence, the available evidence is extremely biased towards the specifics of work environments in healthcare with low generalizability to other workplaces. We must ask ourselves the question whether our research priorities reflect sufficiently societal needs.

Second, the synthesis of evidence presented in the 52 reviews seldom demonstrates that improvements in the psychosocial work environment are causally linked to improvements in workers’ health. In line with the terminology of the authors, it remains to be seen in many interventions whether proximal effects are associated with distal ones. The noticeable exceptions were (1): interventions increasing the influence of workers on work tasks or organization, thus increasing job control, had a positive effect on health and (2) interventions on teamwork, workflow changes, and the like, which resulted in substantial improvement in the work environment that in turn led to a reduction in occurrence of burnout. A linked issue, noted by the authors, is that the quality of the psychosocial work environment may moderate the effectiveness of the intervention. There is certainly a need for more advanced study designs that unravel the mechanism of how and when interventions are effective.

Third, linked to the discussion about the appropriate study design for evaluating effectiveness of interventions in occupational health, in most reviews the majority of original studies had some type of control group, but the use of an RCT design was low. This illustrates the intrinsic difficulty of randomization of workplaces for many different reasons (10). The disadvantages of not being able to conduct a (cluster) RCT should be counteracted with designs for observational studies that offer better insights into the influence of the organizational context and the quality of implementation on successful organizational-level interventions.

The meta-review of Aust and colleagues is not only of interest because of its specific content, it also raises important considerations for intervention research. I propose the following five guiding principles for intervention research on the psychosocial work environment:

## 1. Define the target population and subsequent prevention strategy carefully

In primary prevention strategies, the essential choice is between selective or universal prevention (11). An organizational-level intervention targeting every worker in the company, ie, the universal prevention strategy, should be able to demonstrate a shift in the distribution of risk in the population, eg, flexible working time arrangements are used by a substantial proportion of the workers. Alternatively, selective prevention is more appropriate when particular groups in the company have an increased risk. In such a situation, organizational-level interventions must be much more aligned with the needs of these high-risk groups, eg, teamwork training in units with high sickness absence.

## 2. Choose your outcome measure wisely

In intervention studies with a short follow-up period, it is sensible to focus on proximal endpoints, ie, changes in the psychosocial work environment, rather than distal endpoints such as sickness absence or workers’ health.

## 3. Consider the potential impact of the intervention

When the proportion of workers’ health attributed to the risk factor of interest is modest then the reduction in the risk factor due to the intervention must be large to have a discernable impact on workers’ health. Remember that most workers are quite healthy, thus, large effects in universal interventions cannot be expected.

## 4. Evaluate the pathway of effectiveness of the intervention

We often lack insight into whether an organizational intervention introduces improvements in the psychosocial work environment, and, consequently, that through these improvements the beneficial effects on workers’ health are achieved. Without insight into the pathway of effectiveness, interventions will remain a black box.

## 5. Determine the impact of implementation and context on effectiveness

It is a misconception that the effectiveness of an intervention is primarily determined by its content and quality of delivery. The implementation and specific context in the organization are equally as important. New analytical evaluation methods may be required, acknowledging that individual behavior and circumstances, specific working conditions, and higher-level contextual factors interact continuously.
